# Two new species of *Jalapriya* and a new record, *Dictyocheirosporavinaya* from freshwater habitats in China

**DOI:** 10.3897/BDJ.9.e74295

**Published:** 2021-10-28

**Authors:** Xi Fu, Dan-Feng Bao, Zong-Long Luo, Xiu He, Hong-Yan Su

**Affiliations:** 1 College of Agriculture and Biological Sciences, Dali University, Dali, China College of Agriculture and Biological Sciences, Dali University Dali China; 2 Center of Excellence in Fungal Research, Mae Fah Luang University, Chiang Rai, Thailand Center of Excellence in Fungal Research, Mae Fah Luang University Chiang Rai Thailand; 3 Faculty of Life Science and Technology, Kunming University of Science and Technology, Kunming, China Faculty of Life Science and Technology, Kunming University of Science and Technology Kunming China

**Keywords:** asexual morphs, Dictyosporiaceae, freshwater fungi, phylogeny, taxonomy

## Abstract

**Background:**

Pleosporales is the largest order of Dothideomycetes. In recent years, systematics of Pleosporales have undergone considerable revisions. Dictyosporiaceae is one of the newly established families within this order proposed to accommodate holomorphic saprobic Dothideomycetes. Currently 18 genera are recognised in Dictyosporiaceae.

**New information:**

The new species, *Jalapriyaaquaticum* sp. nov. and *J.apicalivaginatum* sp. nov. were collected from freshwater habitats in Gansu and Yunnan Provinces, China, respectively and are introduced, based on morphology and molecular analysis of combined ITS, LSU, SSU and TEF1-α sequence data. We also recovered one fresh collection of *Dictyocheirosporavinaya* D’souza, Bhat & K.D. Hyde, which is a new record for China. *Jalapriyaaquaticum* differs from extant species of *Jalapriya* in rows converging at the apex and apical cells with spherical-like appendages. *Jalapriyaapicalivaginatum* differs from extant species of *Jalapriya* in having the rows of conidia mostly arranged in a plane. The phylogenetic analysis place the new collections within Dictyosporiaceae (Pleosporales). Descriptions and illustrations of *Jalapriyaaquaticum*, *J.apicalivaginatum* and *Dictyocheirosporavinaya* are provided. A synopsis of characters of species of *Jalapriya* is also provided.

## Introduction

Pleosporales is the largest order of Dothideomycetes. In recent years, various families and genera in the Pleosporales have undergone considerable revisions (Goh and Hyde 1999, Cai et al. 2008, Tanaka et al. 2009, Zhang et al. 2009, Zhang et al. 2012, Hyde et al. 2013, Ariyawansa et al. 2015, Wang et al. 2016). *Boonmee et al. (2016)* accepted eleven genera in the family Dictyosporiaceae (Pleosporales) to accommodate most cheirosporous hyphomycetous genera that are saprobes on decaying wood and plant debris in terrestrial and freshwater habitats. One of the diagnostic characteristics of Dictyosporiaceae is their multicellular cheiroid conidia and this morphological feature distinguishes it from other families in the suborder Massarineae (Hyde et al. 2016). Liu et al. (2017) and Yang et al. (2018) updated the phylogenetic tree for Dictyosporiaceae and introduced two new genera *Aquadictyospora* and *Dendryphiella* in the family. Subsequently, three additional genera, *Neodendryphiella, Pseudoconiothyrium* and *Paradictyocheirospora* were added (Iturrieta-González et al. 2018, Crous et al. 2019, Rajeshkumar et al. 2021). Currently, 18 genera are accepted in Dictyosporiaceae (Boonmee et al. 2016, Li et al. 2017, Iturrieta-González et al. 2018, Yang et al. 2018, Crous et al. 2019, Hyde et al. 2020, Dong et al. 2020, Rajeshkumar et al. 2021).

The genus *Jalapriya* was introduced by Boonmee et al. (2016) with *Jalapriyapulchra* D'souza, Su, Luo & K.D. Hyde as type species; It is characterised by dark brown to black colonies, acrogenous, solitary and cheiroid conidia (Boonmee et al. 2016). Presently, three species are accepted in the genus, *Jalapriyainflata*, *J.pulchra* and *J.toruloides*.

*Dictyocheirospora* was established by Boonmee et al. (2016) to accommodate three new species, *Dictyocheirosporabannica*, *D.rotunda* and *D.vinaya* and four new combinations, *D.gigantica*, *D.heptaspora*, *D.pseudomusae* and *D.subramanianii*. *Dictyocheirospora* is characterised by non-complanate conidia with arms arising from the basal cell and closely gathered at the apex and compact (Wang et al. 2016). The species of *Dictyocheirospora* have been reported from freshwater and terrestrial habitats in China, Japan and Thailand (Jayasiri et al. 2015, Boonmee et al. 2016, Wang et al. 2016, Hyde et al. 2017, Li et al. 2017, Yang et al. 2018, Tibpromma et al. 2018, Phookamsak et al. 2019, Phukhamsakda et al. 2020). Currently, 23 species are accepted in the genus (Boonmee et al. 2016, Yang et al. 2018, Index Fungorum - Search Page).

In this study, two new species *Jalapriyaaquaticum* and *J.apicalivaginatum* and a new geographic record, *Dictyocheirosporavinaya* are introduced, based on morphology and phylogenetic analyses. Detailed descriptions and illustrations are provided.

## Materials and methods

### Isolation and morphological examination

Submerged woody substrates were collected from dynamic waters, Gansu and Yunnan Provinces and taken back to the laboratory in Zip-lock plastic bags. The samples were incubated in plastic boxes lined with moistened tissue paper at room temperature for one week. Methods of morphological observation and isolation follow Luo et al. (2018) and Senanayake et al. (2020).

The pure cultures were developed by single spore isolation following the method provided by Chomnunti et al. (2014). The cultures are deposited in Kunming Institute of Botany, Chinese Academy of Sciences (KUMCC) and China General Microbiological Culture Collection Center (CGMCC). Herbarium specimens are deposited at the Herbarium of Cryptogams Kunming Institute of Botany Academia Sinica (Herb. HKAS). Facesoffungi and Index Fungorum numbers were obtained as in Jayasiri et al. (2015) and Index Fungorum - Search Page.

### DNA extraction, PCR ampliﬁcation and sequencing

Genomic DNA was extracted from fresh mycelia grown on PDA at room temperature. The EZ geneTM Fungal gDNA kit (GD2416) was used to extract DNA according to the manufacturer’s instructions. ITS, LSU, TEF1-α, SSU gene regions were amplified using the primer pairs ITS5/ITS4, LROR/LR5, EF1-983F/EF1-2218R and NS1/NS4. The final volume of the PCR reaction was 25 µl and contained 12.5 µl of 2 × Power Taq PCR MasterMix (a premix and ready-to-use solution, including 0.1 Units/µl Taq DNA Polymerase, 500 µM dNTP Mixture each (dATP, dCTP, dGTP, dTTP), 20 mM Tris– HCl pH 8.3, 100 mM KCl, 3 mM MgCl_2_, stabiliser and enhancer), 1 μl of each primer (10 μM), 1 µl genomic DNA extract and 9.5 µl deionised water. The PCR thermal cycle programme for ITS, LSU, TEF1α and SSU amplification was as follows: initial denaturation of 94°C for 3 minutes, followed by 35 cycles of denaturation at 94°C for 45 seconds, annealing at 56°C for 50 seconds, elongation at 72°C for 1 minute and the final extension at 72°C for 10 minutes. PCR products were purified using minicolumns, purification resin and buffer according to the manufacturer’s protocols (Amershamproduct code: 27–9602–01). The sequencing works were carried by Tsingke Biological Engineering Technology and Services Co. Ltd (Yunnan, P.R. China).

### Phylogenetic analysis

Sequence data for relevant strains were downloaded from GenBank following recent publications (Boonmee et al. 2016, Li et al. 2017, Wang et al. 2016). The consensus sequences were initially aligned using MAFFT v.7 (http://mafft.cbrc.jp/alignment/server/) (Katoh and Standley 2013) and optimised manually when needed. The aligned dataset was analysed by Maximum Likelihood (ML) and Bayesian Inference (BI).

**Figure 1.**
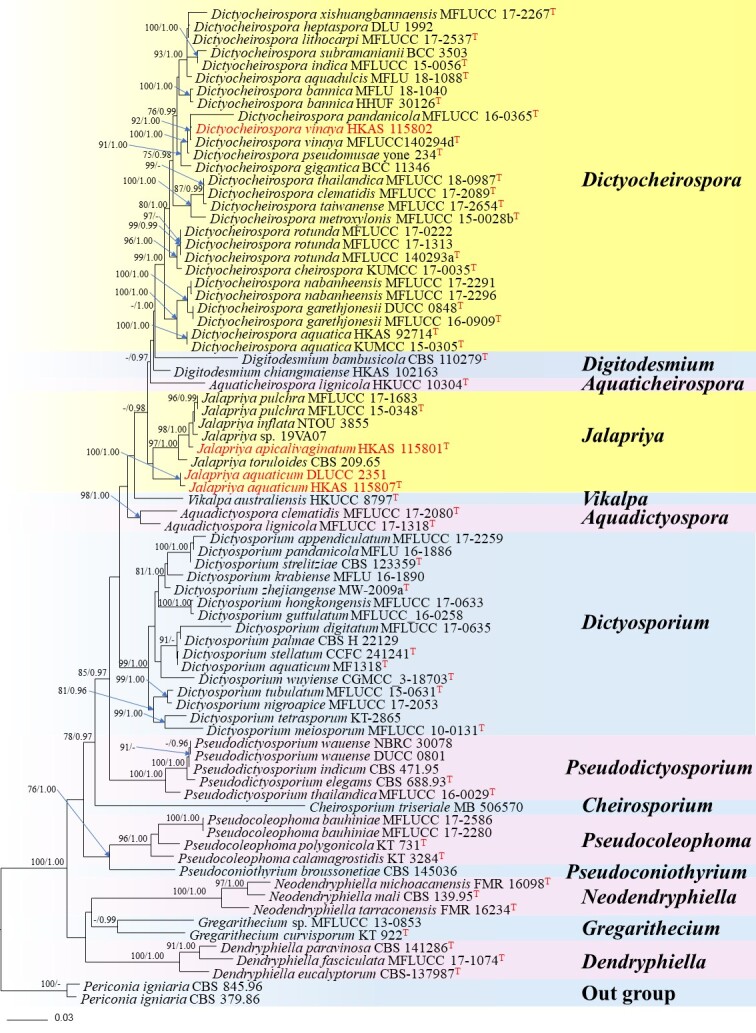
RAxML tree generated from combined LSU, ITS, TEF1-α and SSU sequence data. Bootstrap support values for Maximum Likelihood (the first value) ≥ 75% and Bayesian posterior probabilities (the second value) ≥ 0.95 are placed near the branches as ML/BYPP. The tree is rooted to *Periconiaigniaria* (CBS 379.86 and CBS 845.96). Newly-generated sequences are indicated in red and strains isolated from the holotype and reference specimens are indicated with a red superscript T.

Maximum Likelihood analysis was performed using RAxMLGUI v.1.3 (Silvestro and Michalak 2011). The optimal ML tree search was conducted with 1,000 separate runs using the default algorithm of the programme from a random starting tree for each run. The final tree was selected amongst suboptimal trees from each run by comparing the likelihood scores using the GTR+GAMMA substitution model. Maximum Likelihood bootstrap values equal to or greater than 75% were given as the first set of numbers above the nodes in the resulting ML tree (Fig. 1).

Bayesian analysis was conducted with MrBayes v.3.1.2 (Ronquist and Huelsenbeck 2003) to evaluate posterior probabilities (Rannala and Yang 1996) by Markov Chain Monte Carlo sampling (MCMC). The best-fit models of evolution were estimated by MrModeltest V.2.2 (Nylander and Uppsala University 2004). ITS, LSU and TEF selected the GTR+I+G model with inverse gamma-distributed rate in Bayesian analyses. SSU selected the GTR+G model with inverse gamma-distributed rate in Bayesian analyses. The ML analyses were conducted with RAxML v.7.2.6 (Stamatakis and Alachiotis 2010) using a GTRGAMMA substitution model with 1000 bootstrap replicates. The robustness of the analyses was evaluated by bootstrap support (MLBS). Six simultaneous Markov chains were run for 10 million generations and trees were sampled every 100^th^ generation and 100,000 trees were obtained. The first 20,000 trees, representing the burn-in phase of the analyses, were discarded, while the remaining 80,000 trees were used to calculate posterior probabilities in the majority rule consensus tree (the critical value for the topological convergence diagnostic was 0.01). Through the posterior probabilities (PP) to reflect visually the reliability of each branch without the test for bootstrap method.

The phylogenetic trees were viewed and optimised in FigTree v.1.2.2 (Rambaut and Drummond 2008) and edited further using Microsoft Office PowerPoint. Newly-generated sequences in this study were deposited in GenBank (Table 1).

## Taxon treatments

### Jalapriya apicalivaginatum

D.F. Bao, X. Fu, H.Y. Su & Z.L. Luo, 2021 sp. nov.

BA883ECE-94C7-5EC2-8C20-81539207AF7C

558682

Facesoffungi number: FoF 10257

#### Materials

- **Type status:** Holotype. **Taxon:** scientificName: *Jalapriyaapicalivaginatum*; phylum: Ascomycota; class: Dothideomycetes; order: Pleosporales; family: Dictyosporiaceae; genus: Jalapriya; **Location:** locationRemarks: China, Gansu Province, Gannan City, Xiahe County, Sangke Town, on decaying wood submerged in stream, July 2020; **Event:** habitat: decaying wood submerged in stream; **Record Level:** collectionID: SK 1-21-1 H; collectionCode: L-78

#### Description

**Figure 2.**
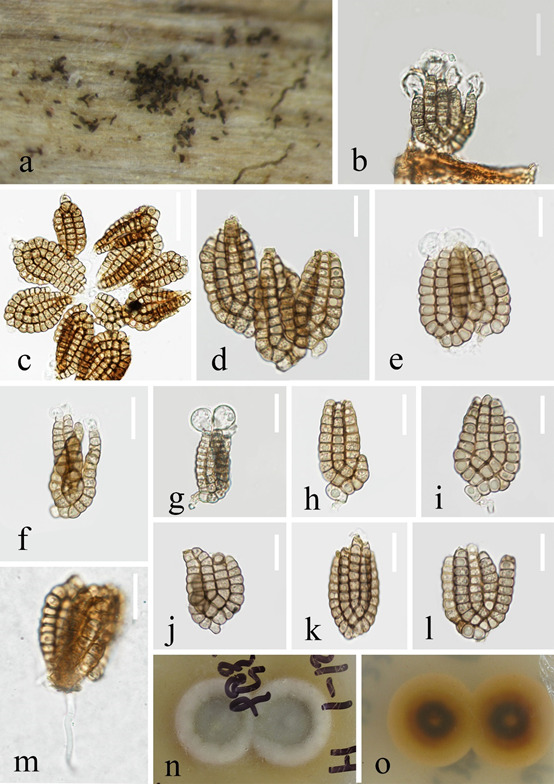
***Jalapriya*** ***apicalivaginatum*** (HKAS 115801, **holotype**). **a** Colonies on submerged wood; **b-l** Conidia; **m** Germinating conidium; **n-o** Culture on PDA from above and reverse. Scale bars: b, f-g, 20 μm; c, 30 μm; d-e, h-m, 15 μm.

Saprobic on decaying wood submerged in stream. **Asexual morph**: Hyphomycetous (Fig. 2). Colonies effuse, scattered, dark brown or black. Mycelium mostly immersed, partly superficial, composed of smooth, septate, branched, hyaline to pale brown hyphae. Conidiophores micronematous, reduced, hyaline to pale brown, unbranched, thin-walled, smooth. Conidiogenous cells holoblastic, integrated, terminal. Conidia acrogenous, solitary, cheiroid, pale brown, the shape of conidia like a "U", with 3–5 rows of cells. The rows in the middle are little bit longer than the outer rows and each row of cells with an apical hyaline, inflated, gelatinous subglobose, cap-like appendage, the rows of conidia mostly arranged in a plane and 2 outer rows arising from a basal cell, rows not separating, each row consisting of 6–12 cells, the size of outer rows 15–52 × 3–6 μm (x̄ = 36 × 5 μm, n = 30), excluding apical hyaline gelatinous appendages, the size of inner rows 24–47 × 4–7 μm (x̄ = 40 × 5.5 μm, n = 30). The size of conidia 24–47 × 17–31.5 µm (x̄ = 40 × 23 μm, n = 30). **Sexual morph**: Undetermined.

##### Culture characteristics

Conidia germinating on PDA within 24 h, germ tubes arising from the outermost cells of the conidium. Colonies on MEA covering 9 cm diam., in 4 weeks at 28°C. On the obverse, the edges are white and the middle is greyish-white. On the reverse, colonies appear pale yellow. Sporulation not observed in culture.

##### Material examined

CHINA, Gansu Province, Gannan City, Xiahe County, Sangke Town, 35°8'9"N, 102°27'11"E, on decaying wood submerged in stream, July 2020, Z.L. Luo, SK 1–21–1 H (HKAS 115801, holotype), ex-type living culture, KUNCC 21-10704 = CGMCC 3.20612.

#### Etymology

Referring to the conidia with an apical mucilaginous sheath.

#### Notes

In the phylogenetic analysis, *J.apicalivaginatum* formed a distinct lineage within *Jalapriya* and close to *Jalapriya* sp. (19VA07); However, the morphology of *Jalapriya* sp. (19VA07) was not available, but phylogeny of *J.apicalivaginatum* and *Jalapriya* sp. are distinct. *Jalapriyaapicalivaginatum* resembles *J.pulchra* and *J.inflata* in having each conidial row of cells with an apical hyaline, inflated, gelatinous subglobose, cap-like appendage. However, *Jalapriyainflata* is characterised by branched conidiophores, whereas conidiophores of *J.apicalivaginatum* are not differentiated. *Jalapriyaapicalivaginatum* has fewer number of rows than those of *J.pulchra* (3–5 rows vs. 5–7 rows) and conidia are smaller than those of *J.pulchra* (24–47 × 17–31.5 µm vs. 32–46 × 23.5–31.5 μm) (Boonmee et al. 2016) (Table 2).

### Jalapriya aquaticum

D.F. Bao, X. Fu, H.Y. Su & Z.L. Luo, 2021 sp. nov.

DA9CA5AF-A62A-536C-B1FF-3F64EC6DE84F

558683

Facesoffungi number: FoF 10258

#### Materials

- **Type status:** Holotype. **Taxon:** scientificName: *Jalapriyaaquaticum*; phylum: Ascomycota; class: Dothideomycetes; order: Pleosporales; family: Dictyosporiaceae; genus: Jalapriya; **Location:** locationRemarks: China, Yunnan Province, Dali, Cangshan Mountain, Lingquan stream, on decaying wood submerged in stream, April 2019; **Event:** habitat: Saprobic on decaying wood submerged in stream; **Record Level:** collectionID: 1LQX III H Z-7-1; collectionCode: S-2101

#### Description

**Figure 3.**
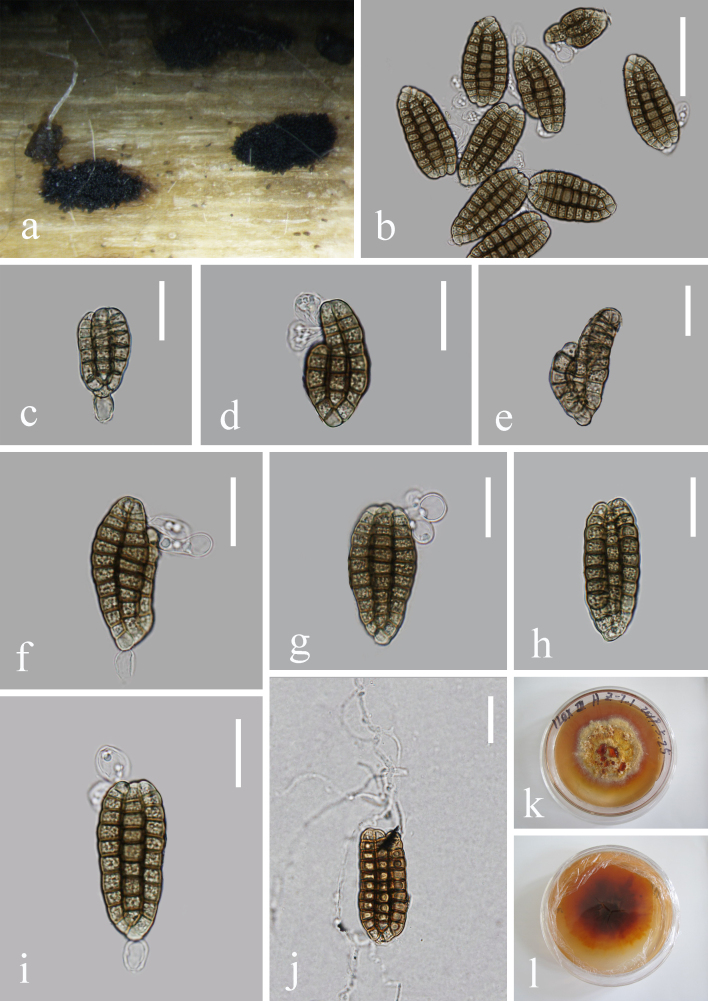
***Jalapriyaaquaticum*** (HKAS 115807, **holotype**). **a** Colonies on submerged wood; **b** Squash mount of conidioma; **c-i** Conidia; **j** Germinating conidium; **k-l** Culture on PDA from above and reverse. Scale bars: b, 40 μm; c-j, 20 μm.

Saprobic on decaying wood submerged in stream. **Asexual morph**: Hyphomycetous (Fig. 3). Colonies punctiform, sporodochial, velvety, dark brown to black. Conidiophores micronematous, subhyaline to pale brown hyphae, unbranched, thin-walled, smooth. Mycelium immersed, composed of brown, smooth, thin-walled, septate. Conidiogenous cells holoblastic, integrated, terminal. Conidia acrogenous, solitary, cheiroid, pale to medium brown, with 3–4 rows of cells, rows converging at apex, apical cells with spherical-like appendages, the immature conidia are slightly curved and become straight after maturity. Two outer rows arising from a basal cell, rows not separating, each row consisting of 6–12 cells, the size of outer rows 29–53 × 6–8 μm (x̄ =45 × 5 μm, n = 30), excluding apical hyaline gelatinous appendages, the size of inner rows 22–44 × 4–8 μm (x̄ = 38 ×6 μm, n = 30). The size of conidia 22–53 × 16–24 µm. **Sexual morph**: Undetermined.

##### Culture characteristics

Conidia germinating on PDA within 24 h, germ tubes arising from the outermost cells of the conidium. Colonies on MEA covering 9 cm diam., in 4 weeks, at 28°C, white to cream. Sporulation not observed in culture.

##### Material examined

CHINA, Yunnan Province, Dali, Cangshan Mountain, Lingquan stream, 25.747501°N, 100.090989°E, on decaying wood submerged in stream, April 2019, Z.Q. Zhang, 1LQX III H Z-7-1 (S-2101) (HKAS 115807, holotype), ex-type living culture, KUNCC 21-10705 = DLUCC 2101 = CGMCC 3.20613; ibid. July 2019, Zhengquan Zhang, 2LQX III Z-56-1 H (S-2351), living culture, KUNCC 21-10706 = DLUCC 2101.

#### Etymology

Referring to the species collected from aquatic habitats.

#### Notes

In the phylogenetic analysis, *J.aquaticum* nested in *Jalapriya* and sister to *J.toruloides*. Morphologically, *J.aquaticum* is similar to *J.* *inflata* in having 3–4 rows of conidia, but differs from *J.* *inflat* in the shape of the conidia, the cells of *J.inflata* are fuller and more three-dimensional. *J.inflata* arranged more loosely in the rows of conidia and *J.aquaticum* packed more tightly. *J.aquaticum* has larger conidia than those of *J.inflata* (22–53 × 16–24 vs. 28.5–38 × 14.5–21.5 μm). *Jalapriyaquaticum* similar to *J.* *pulchra* in having appendages on the apical cells of the conidia, but differs in the rows of *J.aquaticum* not being separable without manual force.

### Dictyocheirospora vinaya

D’souza, Bhat & K.D. Hyde, 2016, Fungal Diversity 80: 465

3F9E5904-C9A7-52E9-80F5-561ABF37CEB2

Facesoffungi number: FoF 01263

#### Materials

- **Type status:** Holotype. **Taxon:** scientificName: *Dictyocheirosporavinaya*; phylum: Ascomycota; class: Dothideomycetes; order: Pleosporales; family: Dictyosporiaceae; genus: Dictyocheirospora; **Location:** locationRemarks: Thailand. Chiang Mai, Mushroom Research Centre, on submerged wood in a freshwater stream, 24 November 2013; **Identification:** identifiedBy: D’souza, Bhat & K.D. Hyde; **Event:** habitat: submerged wood in a freshwater stream; **Record Level:** type: MFLU 14–0264; collectionCode: MJD-26; source: https://doi.org/10.1007/s13225-016-0363-z

#### Description

**Figure 4.**
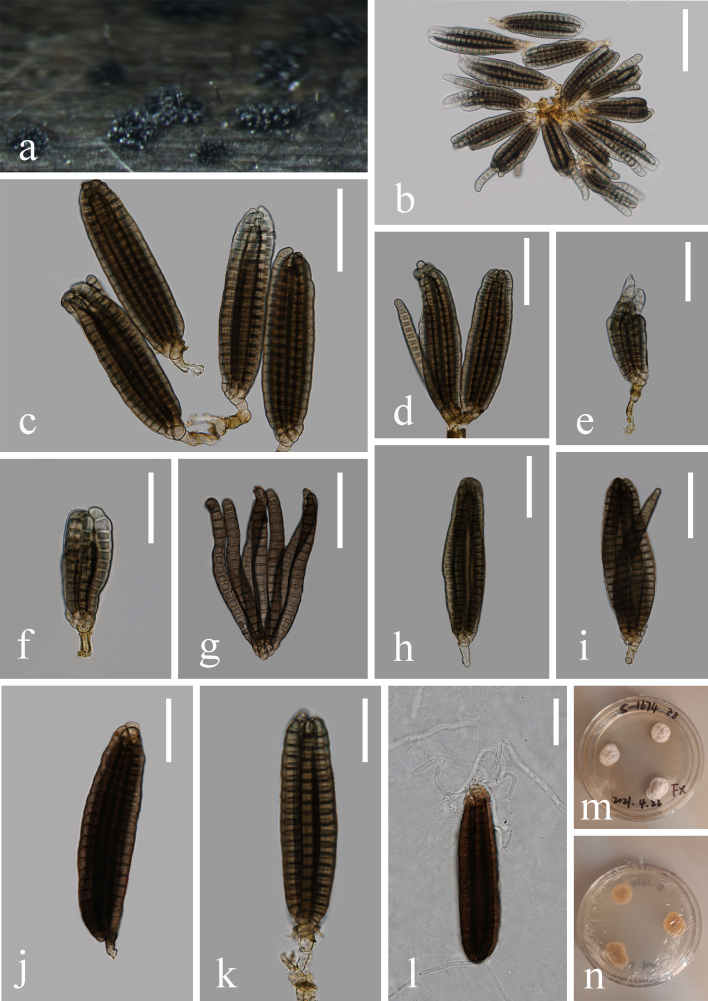
***Dictyocheirosporavinaya*** (HKAS 115802). **a** Colonies on submerged wood; **b** Squash mount of conidioma; **c-k** Conidia; **l** Germinating conidium; **m-n** Culture on PDA from above and reverse. Scale bars: b, 50 μm; c-d, h-k, 40 μm; e-g, l, 30 μm.

Saprobic on decaying wood in streams. **Asexual morph**: Hyphomycetous (Fig. 4). Colonies punctiform, sporodochial, velvety, dark brown. Mycelium immersed, composed of pale brown, smooth, thin-walled septate, branched, 1–2 μm wide hyphae. Conidiophores 9–27 × 3–6 μm (x̄ = 15 × 5 μm, n = 18), micronematous to semi-macronematous, pale brown, smooth, thin-walled. Conidiogenous cells holoblastic, integrated, terminal, determinate, pale brown. Conidia solitary, terminal, cheiroid, 48–110 × 14–32 μm (x̄ = 73 × 22 μm, n = 30), pale brown, consisting of 7 rows of cells; rows digitate, arising from a basal cell, each arm consisting of 10–20 cells, distoseptate, constricted at septa, rows appressed when young, inwardly curved at the tip, palmately divergent when squashed, smooth-walled, guttulate. **Sexual morph**: Undetermined.

##### Culture characteristics

Conidia germinating on water agar within 24 h, germ tubes emerging from the basal cells of the conidium. Colonies on PDA covering 9 cm diam., in 4 weeks, at 28°C, with wavy margins, at first white, later becoming orange. Sporulating regions scattered, but mostly confined to the centre of the culture.

##### Material examined

CHINA, Yunnan Province, Nanpanjiang River, 24°33'57.48"N, 103°06'44.44"E, on decaying wood submerged in stream, 23 February 2018, X. He, NPJ H 3–2–1 (HKAS 115802); living culture KUNCC 21-10707.

#### Notes

*Dictyocheirosporavinaya*, the type species of *Dictyocheirospora*, was introduced by Boonmee et al. (2016). *Dictyocheirosporavinaya* is characterised by punctiform, dark brown colonies, pale brown, solitary, terminal, cheiroid conidia. Our fresh collection fits perfectly with the original description of *D.vinaya* (Boonmee et al. 2016). Phylogenetic analyses showed that our strain (DLUCC 1674) clustered with the ex-type strain of *D.vinaya* with high bootstrap support (93% ML and 1.00 PP). ITS comparison between our strain and MFLUCC 14–0294 revealed that there is no difference in a total of 499 bp, comparison of LSU between our strain and MFLUCC 14–0294 revealed 3 bp differences in a total of 1252 bp. Thus, we identified our new collection as *D.vinaya*, based on both phylogeny and morphology. *Dictyocheirosporavinaya* MFLUCC 14–0294 collected from freshwater habitats in Thailand, while our new collection was collected from freshwater habitats in China. It is a new record for China.

## Identification Keys

### Key to *Jalapriya* species

**Table:** 

1	Conidia without appendages	*J.toruloides*
–	Conidia with appendages	3
2	Conidia composed of 5–7 rows	*J.pulchra*
–	Conidia composed of 3–5 rows	3
3	Conidia 28.5–38 × 14.5–21.5 μm	*J.inflata*
–	The size of conidia not as above	4
4	Apical cell of conidia with spherical-like appendages	*J.aquaticum*
–	Apical cell of conidia with cap-like appendages	*J.apicalivaginatum*

## Analysis

**Phylogenetic analysis**

The combined ITS, LSU, TEF1-α and SSU dataset consisted 78 sequences representing all genera of the Dictyosporiaceae with *Periconiaigniaria* (CBS 379.86 and CBS 845.96) as outgroup taxon. The best scoring RaxML tree with the final ML optimisation likelihood value of –20943.450686 is shown here (Fig. 1). The alignment comprised 4309 characters including gaps. The matrix had 1309 distinct alignment patterns, with 51.74% undetermined characters or gaps. Estimated base frequencies were as follows: A = 0.241918, C = 0.244332, G = 0.269566, T = 0.244184; substitution rates AC = 1.667496, AG = 3.298982, AT = 2.345910, CG = 0.903092, CT = 8.345950, GT = 1.000000; Tree-Length = 1.761576.

Two newly-collected *Jalapriyaaquaticum* isolates grouped with *species of Jalapriya* and basal to the genus with highly-supported value (100 ML/1.00 PP). *Jalapriyaapicalivaginatum* formed a distinct lineage between *J.toruloides* and *Jalapriya* sp. (19VA07) with high bootstrap (97 ML/1.00 PP). *Dictyocheirosporavinaya* (HKAS 115802) clustered with its ex-type strains with high support (93 ML/1.00 PP).

## Discussion

Dictyosporiaceae accommodates a holomorphic group of Dothideomycetes, including 18 genera (Hyde et al. 2019, Rajeshkumar et al. 2021). *Dictyocheirospora* is the second largest genus of Dictyosporiaceae, followed by *Dictyosporium*. *Dictyocheirospora* is morphologically similar to *Dictyosporium* in having cheiroid, cylindrical conidia; However, *Dictyocheirospora* differs from *Dictyosporium* in having non-complanate conidia with arms arising from the basal cell and closely gathered at the apex and compact, while *Dictyosporium* has complanate conidia without separating arms. Thus, eight species were transferred from *Dictyosporium* to *Dictyocheirospora*, based on themorphological characters and phylogenetic analyses. (Boonmee et al. 2016, Yang et al. 2018). *Dictyocheirospora* is cosmopolitan in distribution and commonly reported from freshwater habitats in China, India, Japan and Thailand. Nine species of *Dictyocheirospora* were found on submerged decaying wood, others were found in terrestrial habitats. Currently, nine species have been discovered in China including *Dictyocheirosporavinaya*, which is mentioned in this article. (Boonmee et al. 2016, Wang et al. 2016, Hyde et al. 2017, Li et al. 2017, Yang et al. 2018, Tibpromma et al. 2018, Jayasiri et al. 2015, Phookamsak et al. 2019, Phukhamsakda et al. 2020, Rajeshkumar et al. 2021).

Currently, three species are accepted in *Jalapriya*, of which, *J.toruloides* (Corda) is a terrestrial species discovered by Henningsson (1974) in Sweden; Afterwards it has been found in subtropical to temperate areas of both hemispheres, seemingly more often reported from coastal localities, considered an euryhaline species (Tibell et al. 2020), but our fresh collections are all from submerged wood in freshwater lotic habitats. In addition, both *J.pulchra* and *J.acuaticum* were all found in Yunnan Province, China on decaying wood submerged in a stream (Table 2). The morphological differences between *J.apicalivaginatum* and *J.pulchra* are not significant, but they are phylogenetically distinct. Morphology of *J.toruloides* is not available; However, the new species *J.aquaticum* forms a distinct clade from *J.toruloides*. *Jalapriyaaquaticum* is different from other species in *Jalapriya* and forms a separate branch with high support value (100% ML and 1.00 BYPP).

## Supplementary Material

XML Treatment for Jalapriya apicalivaginatum

XML Treatment for Jalapriya aquaticum

XML Treatment for Dictyocheirospora vinaya

## Figures and Tables

**Table 1.** Isolates and sequences used in this study (newly-generated sequences are indicated in bold, strains isolated from the holotype and reference specimens are indicated in with a T, without GenBank accession numbers are indicated in "_") .

**Taxon**	**Voucher/culture**		**GenBank accession numbers**			
			**ITS**	**LSU**	**TEF1α**	**SSU**
*Aquaticheirosporalignicola*		HKUCC 10304^T^	AY864770	AY736378	_	AY736377
*Aquadictyosporaclematidis*		MFLUCC 17-2080^T^	NR171871	_	MT394727	NG070646
*A.lignicola*		MFLUCC 17-1318^T^	MF948621	MF948629	MF953164	_
*Cheirosporiumtriseriale*		MB 506570	EU413953	EU413954	_	_
*Dendryphiellaeucalyptorum*		CBS 137987^T^	KJ869139	KJ869196	_	_
*Den.fasciculata*		MFLUCC 17-1074^T^	MF399213	MF399214	_	_
*Den.paravinosa*		CBS 141286^T^	KX228257	KX228309	_	_
*Dictyocheirosporaaquadulcis*		MFLU 18-1088^T^	MK634545	MK634542	_	_
*Di.aquatica*		KUMCC 15-0305^T^	KY320508	KY320513	_	_
*Di.aquatica*		HKAS 92714^T^	NR154030	_	_	_
*Di.bannica*		HHUF 30126^T^	NR154039	NG059061	AB808489	NG064841
*Di.bannica*		MFLU 18-1040	MH381765	MH381774	_	MH381759
*Di.cheirospora*		KUMCC 17-0035^T^	MF177035	MF177036	_	MF928073
*Di.clematidis*		MFLUCC 17-2089^T^	MT310593	MT214546	MT394728	MT226665
*Di.garethjonesii*		MFLUCC 16-0909^T^	KY320509	KY320514	_	_
*Di.garethjonesii*		DUCC 0848^T^	MF948623	MF948631	MF953166	_
*Di.gigantica*		BCC 11346	DQ018095	_	_	_
*Di.heptaspora*		DLU 1992	MT756244	MT756243	_	_
*Di.indica*		MFLUCC 15-0056^T^	MH381763	MH381772	MH388817	MH381757
*Di.lithocarpi*		MFLUCC 17-2537^T^	NR163345	NG070074	_	NG065783
*Di.metroxylonis*		MFLUCC 15-0028b^T^	MH742322	MH742314	MH764303	MH742318
*Di.nabanheensis*		MFLUCC 17-2291	MK347748	MK347965	MK360050	_
*Di.nabanheensis*		MFLUCC 17-2296	MK347756	MK347973	MK360051	_
*Di.pandanicola*		MFLUCC 16-0365^T^	MH388341	MH376713	MH388376	_
*Di.pseudomusae*		Yone 234^T^	LC014550	AB807520	AB808496	AB797230
*Di.rotunda*		MFLUCC 17-0222	MH381764	MH381773	MH388818	MH381758
*Di.rotunda*		MFLUCC 140293a^T^	KU179099	KU179100	_	_
*Di.rotunda*		MFLUCC 17-1313	MF948625	MF948633	MF953168	_
*Di.subramanianii*		BCC 3503	DQ018094	AB807520	_	_
*Di.taiwanense*		MFLUCC 17-2654^T^	MK495821	MK495820	_	_
*Di.thailandica*		MFLUCC 18-0987^T^	NR171885	MN913743	_	_
*Di.vinaya*		MFLUCC140294d^T^	KU179102	KU179103	_	KU179104
***Di.vinaya***		**HKAS 115802**	**MZ618659**	**MZ618660**	**MZ851994**	**_**
*Di.xishuangbannaensis*		MFLUCC 17-2267^T^	MH388342	MH376714	MH388377	_
*Dictyosporiumappendiculatum*		MFLUCC 17-2259	MH388343	MH376715	_	_
*Dictyos.aquaticum*		MF1318^T^	KM610236	_	_	_
*Dictyos.digitatum*		MFLUCC 17-0635	MH388344	MH376716	MH388378	_
*Dictyos.guttulatum*		MFLUCC 16-0258	MH388345	MH376717	MH388379	MH388312
*Dictyos.hongkongensis*		MFLUCC 17-0633	MH388346	MH376718	MH388380	NG068388
*Dictyos.meiosporum*		MFLUCC 10-0131^T^	KP710944	KP710945	_	_
*Dictyos.nigroapice*		MFLUCC 17-2053	MH381768	MH381777	MH388821	_
*Dictyos.krabiense*		MFLU 16-1890	_	MH376719	MH388381	_
*Dictyos.palmae*		CBS H-22129	_	KX555648	_	_
*Dictyos.pandanicola*		MFLU 16-1886	MH388347	MH376720	MH388382	_
*Dictyos.stellatum*		CCFC 241241^T^	NR154608	JF951177	_	_
*Dictyos.strelitziae*		CBS 123359^T^	NR156216	FJ839653	_	_
*Dictyos.tetrasporum*		KT 2865	LC014551	AB807519	AB808495	_
*Dictyos.tubulatum*		MFLUCC 15-0631^T^	MH381769	MH381778	MH388822	_
*Dictyos.wuyiense*		CGMCC 3-18703^T^	KY072977	_	_	_
*Dictyos.zhejiangense*		MW-2009a^T^	FJ456893	_	_	_
*Dictyos.bambusicola*		CBS 110279^T^	DQ018091	DQ018103	_	_
*Dictyos.chiangmaiense*		HKAS 102163	_	MK571766	_	MK571775
*Gregaritheciumcurvisporum*		KT 922^T^	AB809644	AB807547	_	AB797257
*Gregarithecium* sp.		MFLUCC 13-0853	KX364281	KX364282	_	KX364283
***Jalapriyaapicalivaginatum***		**HKAS 115801^T^**	**MZ621167**	**MZ621168**	_	_
***J.aquaticum* (2101)**		**HKAS 115807^T^**	**MZ621152**	**MZ621169**	**MZ851995**	**MZ621170**
***J.aquaticum* (2351)**		**DLUCC 2351**	**MZ621151**	**MZ621165**	_	**MZ621166**
*J.inflata*		NTOU 3855	JQ267362	JQ267363	_	JQ267361
*J.pulchra*		MFLUCC 15-0348^T^	KU179108	KU179109	_	KU179110
*J.pulchra*		MFLUCC 17-1683	MF948628	MF948636	MF953171	_
*Jalapriya* sp.		19VA07	JX270548	_	_	_
*J.toruloides*		CBS 209.65	DQ018093	DQ018104	_	DQ018081
*Neodendryphiellamali*		CBS 139.95^T^	LT906655	LT906657	_	_
*N.michoacanensis*		FMR 16098^T^	LT906660	LT906658	_	_
*N.tarraconensis*		FMR 16234^T^	LT906659	LT906656	_	_
*Periconiaigniaria*		CBS 379.86	LC014585	AB807566	AB808542	AB797276
*P.igniaria*		CBS 845.96	LC014586	AB807567	AB808543	GU296171
*Pseudocoleophomabauhiniae*		MFLUCC 17-2280	MK347735	MK347952	MK360075	MK347843
*Pseudoc.bauhiniae*		MFLUCC 17-2586	MK347736	MK347953	MK360076	MK347844
*Pseudoc.calamagrostidis*		KT 3284^T^	LC014592	LC014609	LC014614	LC014604
*Pseudoc.polygonicola*		KT 731^T^	AB809634	AB807546	AB808522	AB797256
*Pseudoc.typhicola*		MFLUCC 16-0123^T^	KX576655	KX576656	_	_
*Pseudoconiothyriumbroussonetiae*		CBS 145036	MK442618	MK442554	MK442709	_
*Pseudodictyosporiumelegams*		CBS 688.93^T^	MH862454	MH874101	_	DQ018084
*Pseudodi.indicum*		CBS 471.95	DQ018097	_	_	_
*Pseudodi.thailandica*		MFLUCC 16-0029^T^	KX259520	KX259522	KX259526	KX259524
*Pseudodi.wauense*		NBRC 30078	DQ018098	DQ018105	_	DQ018083
*Pseudodi.wauense*		DUCC 0801	MF948622	MF948630	MF953165	_
*Vikalpaaustraliensis*		HKUCC 8797^T^	DQ018092	_	_	_

**Table 2.** A synopsis of characters of species of *Jalapriya*.

**Species**	**Conidia**				**Distribution**	**Reference**
	**Shape**	**Size (μm)**	**Colour**	**Number of rows**		
*Jalapriyainflata*	Euseptate, thin-walled and staurosporous, composed of an apically inflated basal cell	28.5–38 × 14.5–21.5	Brown	3–4 rows	UK, Ontario, On rotten wood	Matsushima 1983, Kirschner et al. 2013, Boonmee et al. 2016, Iturrieta-González et al. 2018
*J.pulchra*	Acrogenous, solitary, each row of cells with an apical hyaline, inflated, gelatinous subglobose, cap-like appendage	32–46 × 23.5–31.5	Uniformly pale to medium reddish-brown	5–7 rows	CHINA, Yunnan Province, on decaying wood submerged in stream	Boonmee et al. 2016, Iturrieta-González et al. 2018
*J.aquaticum*	Acrogenous, solitary, rows converging at apex, apical cells with spherical-like appendages	22–53 × 16–24	Pale to medium brown	3–4 rows	CHINA, Yunnan Province, on decaying wood submerged in stream	This study
*J.apicalivaginatum*	Acrogenous, solitary, thin-walled, each row of cells with an apical hyaline, inflated, gelatinous subglobose, cap-like appendage	24–47 × 17–31.5	pale brown	3–5 rows	CHINA, Gansu Province, on decaying wood submerged in stream	This study
